# Differential Genetic Effect of the Norepinephrine Transporter Promoter Polymorphisms on Attention Problems in Clinical and Non-clinical Samples

**DOI:** 10.3389/fnins.2018.01051

**Published:** 2019-01-14

**Authors:** Zsofia Nemoda, Nora Angyal, Zsanett Tarnok, Emma Birkas, Emese Bognar, Maria Sasvari-Szekely, Judit Gervai, Krisztina Lakatos

**Affiliations:** ^1^Department of Medical Chemistry, Molecular Biology and Pathobiochemistry, Semmelweis University, Budapest, Hungary; ^2^Vadaskert Child and Adolescent Psychiatric Clinic, Budapest, Hungary; ^3^Institute of Cognitive Neuroscience and Psychology, Research Centre for Natural Sciences, Hungarian Academy of Sciences, Budapest, Hungary; ^4^Institute of Behavioural Sciences, Semmelweis University, Budapest, Hungary

**Keywords:** catecholamine, noradrenaline, SLC6A2 (solute carrier family 6, member 2), ADHD (attention deficit hyperactivity disorder), inattention

## Abstract

Among the monoaminergic modulatory neurotransmitters, norepinephrine is involved in task orienting, hence noradrenergic genetic variants have been studied in connection to attentional processes. The role of this catecholamine system is also highlighted by the selective norepinephrine transporter blocking atomoxetine, which has proved to be effective in the pharmacological treatment of Attention Deficit Hyperactivity Disorder (ADHD). In the present genetic association study three single nucleotide polymorphisms (rs28386840, rs2242446, rs3785143 SNPs) were analyzed from the 5′ region of the norepinephrine transporter (*NET*, *SLC6A2*) gene, which have been linked to ADHD previously. Attention problems scores of the mother-rated Child Behavior Checklist (CBCL) were used in separate analyses of 88 preschoolers (59.1% male, 6 years of age) recruited from the general population and 120 child psychiatry patients with ADHD diagnosis (85.8% male, age: 9.8 ± 2.9). The *NET* SNPs showed associations with attention problems, but the direction was different in the two groups. Regarding the promoter variant rs28386840, which showed the most consistent association, the T-allele-carrier patients with ADHD had lower CBCL attention problems scores compared to patients with AA genotype (*p* = 0.023), whereas T-allele-carriers in the community sample had more attention problems (*p* = 0.042). Based on previous reports of lower NE levels in ADHD children and the inverted-U shape effect of NE on cognitive functions, we propose that rs28386840 (-3081) T-allele, which is associated with lower NET expression (and potentially higher synaptic NE level) would support attention processes among ADHD patients (similarly as atomoxetine increases NE levels), whereas it would hinder cortical functions in healthy children.

## Introduction

Attention problems have gained increasing interest during the last decades, as the proportion of children with attention deficit hyperactivity disorder (ADHD) diagnosis has risen dramatically in many countries, creating social and scientific debates (Singh, 2008). Although the prevalence of ADHD increased in Western societies, the worldwide prevalence seems to be a stable 5–6% among school-age children (Polanczyk et al., 2014). Therefore, identifying potential risk and protective factors at an early age could help developing preventive strategies. Since both ADHD diagnosis and attention problems show substantial genetic background with complex inheritance, searching for genetic markers has been in the center of many studies.

Importantly, heritability estimates of complex traits vary widely from childhood to adulthood (Polderman et al., 2015). Twin studies of children using parent or teacher ratings reported high heritability estimates (h^2^ ∼ 0.7) for attention problems (Chang et al., 2013; Kan et al., 2013). Heritability estimates of attention problems based on self-report questionnaires decrease in adolescents and adults (h^2^ ∼ 0.4–0.5, Kan et al., 2013). Clearly, there is a substantial effect of the assessment method (see examples listed by Faraone and Larsson, 2018), but the underlying mechanisms may also change during development (Chang et al., 2013). Therefore, our aim was to identify genetic factor(s) of attention problems using a mother-rated symptom scale in a community sample of children in addition to child psychiatry patients, because childhood is potentially the most sensitive period to detect genetic effects.

Attention is often modeled as separate networks responsible for alerting, orienting, and executive control, which are linked to specific neurotransmitter systems (Raz and Buhle, 2006). The norepinephrine (NE) system projects to various cortical areas and functions mostly in alerting, whereas the mesocortical dopamine system is involved in executive control. For optimal cognitive functioning appropriate levels of catecholamine (dopamine and NE) transmitters were proposed, since both lower and higher tone of catecholamines in the prefrontal cortex (PFC) can worsen performance (Berridge and Arnsten, 2013), resulting in inverted-U shaped modulatory effects of these catecholamines. Therefore, association studies trying to identify genetic variations of attention phenotypes have been focusing on catecholamine neurotransmitter systems.

Recently, we have reported genetic associations of single nucleotide polymorphisms (SNPs) of the norepinephrine transporter gene (*NET, SLC6A2*) with ADHD symptom severity but not with ADHD diagnosis *per se* (Angyal et al., 2018). In the present study, we wanted to test if this genetic association could be extended to a non-clinical range of inattention. Therefore, we assessed attention problems with a widely used parent-rated symptom list in both clinical and community samples. Polymorphisms from the 5′ end of the *NET* gene were chosen based on their previous associations with ADHD-related phenotypes in different ethnic groups (Joung et al., 2010; Sengupta et al., 2012; Hohmann et al., 2015). Importantly, these SNPs were in high (but not complete) linkage in the previously studied Hungarian population (Angyal et al., 2018). The promoter SNPs rs28386840 (-3081 A/T) and rs2242446 (-182 T/C) can potentially influence gene expression (Zill et al., 2002; Kim et al., 2006; Sigurdardottir et al., 2016), hence can have functional consequences. A recent brain imaging study showed differential genetic effects of these *NET* promoter variants on transporter density in ADHD patients and controls (Sigurdardottir et al., 2016). Therefore, we conducted the symptom-scale based genetic association analyses separately in the clinical and community samples. Case-control analyses were not run for these samples, because larger ADHD and control groups were compared earlier in our meta-analysis of *NET* polymorphisms (Angyal et al., 2018).

## Methods

The study was designed in compliance with the Helsinki Declaration and was approved by the Local Scientific and Research Ethics Committee of the Hungarian Medical Research Council. The participating parents (mostly mothers) provided written informed consent. The two samples and genotyping methods are described in details by Angyal et al. (2018) and Birkas et al. (2006). Briefly, DNA was isolated from buccal swabs, and *NET* SNPs were genotyped with pre-designed TaqMan probes (rs28386840: C__60398891_10, rs2242446: C__26354911_10, rs3785143: C__27481932_10) on 7300 Real-Time PCR System (Applied BioSystem). No significant deviations from Hardy–Weinberg equilibrium (*p* > 0.1) were detected for the *NET* polymorphisms in any of the tested samples. Both the clinical and the community samples were ethnically homogeneous Caucasian origin and consisted of unrelated individuals.

For psychiatric symptom assessment, the Hungarian version of the Child Behavior Checklist (CBCL, Achenbach, 1991; Gadoros, 1996) was used, applying the standardized T-scores, as these were corrected for sex and age differences. CBCL was available for 88 children in the community sample (mean age: 6.3 ± 0.2 years, 59.1% boys). In the clinical sample, 120 patients (mean age: 9.8 ± 2.9 years, 85.8% boys) had ADHD according to DSM-IV criteria (American Psychiatric Association, 1994) either as primary or secondary diagnosis. Additional 72 patients diagnosed with tic-disorders (but not with ADHD) had CBCL data, yielding a total number of 192 patients comprising an extended child psychiatry patient sample (mean age: 10.0 ± 3.2 years, 81.3% boys). Comorbid conditions were assessed by the Hungarian child version of the Mini-International Neuropsychiatric Interview (MINI-Kid; Balazs et al., 2004). Among the 120 patients with ADHD, 30% had Tourette syndrome, 14.2% obsessive compulsive disorder, 27.5% learning disorder, 23.3% conduct disorder, and 14.2% anxiety disorder. In the extended child psychiatry sample, 62.5% had ADHD, 35.4% Tourette syndrome, 34.4% obsessive compulsive disorder, 19.3% learning disorder, 16.1% conduct disorder, and 26.0% anxiety disorder.

Statistical analyses were carried out with SPSS 20 for Windows, using the T-score of the CBCL attention problems scale as dependent variable and the genotype categories (main allele homozygotes vs. minor allele carriers) as independent variable, with sex and age covariates in univariate analysis of variance in the clinical samples. Whereas CBCL T-scores were compared between the two genotype groups by Mann–Whitney *U*-tests in the community sample. Quantitative analyses of estimated haplotypes were performed with the THESIAS program (Tregouet and Garelle, 2007).

## Results

Genetic associations of the CBCL attention problems were tested separately in the community and the patient samples (Table 1). In these quantitative analyses the rare homozygote and heterozygote genotypes were grouped together to increase statistical power. In the community sample, the promoter rs28386840 and the intronic rs3785143 showed nominally significant associations with attention problems (*Z* = –2.03, *p* = 0.042, and *Z* = –1.97, *p* = 0.049, respectively). Among patients with ADHD, the two promoter SNPs showed associations with attention problems (rs28386840: *F*(1,116) = 5.33, *p* = 0.023, η^2^= 0.04, observed power: 0.63; rs2242446: *F*(1,116) = 5.53, *p* = 0.020, η^2^= 0.05, observed power: 0.64). Similar associations (with higher power) were detected in the extended child psychiatry patient sample: rs28386840: *F*(1,188) = 11.55, *p* = 0.001, η^2^= 0.06, observed power: 0.92; rs2242446: *F*(1,188) = 9.40, *p* = 0.002, η^2^= 0.05, observed power: 0.86). Importantly, the means of the genotype groups showed different patterns in the clinical and community samples (Table 1).

**Table 1:** CBCL attention problems scores according to the NET genotypes in the community and clinical samples.

	Community sample *N* = 88		Patients with ADHD *N* = 120		Child psychiatry patients *N* = 192	
	*N*	Mean ± SD	*N*	Mean ± SD	*N*	Mean ± SD
**rs28386840 (-3081 A/T)**
**AA**	**40**	**55.80 ± 6.32**	**71**	**72.72 ± 8.38**	**106**	**70.61 ± 9.31**
AT	39	59.10 ± 8.31	43	69.49 ± 7.78	75	66.16 ± 9.16
TT	9	59.00 ± 7.43	6	67.33 ± 9.61	11	66.0 ± 10.95
**AT + TT**	**48**	**59.08 ± 8.08**	**49**	**69.22 ± 7.94**	**86**	**66.14 ± 9.33**

*p*-value	0.042		0.023		0.001	

**rs2242446 (-182 T/C)**
**TT**	**40**	**56.05 ± 6.75**	**68**	**72.74 ± 8.55**	**100**	**70.45 ± 9.46**
CT	39	58.85 ± 8.05	43	70.19 ± 7.50	77	66.90 ± 9.28
CC	9	59.00 ± 7.43	9	65.67 ± 8.38	15	65.13 ± 9.64
**CT + CC**	**48**	**58.88 ± 7.86**	**52**	**69.40 ± 7.77**	**92**	**66.61 ± 9.31**

*p*-value	0.064		0.020		0.002	

**rs3785143 (intronic C/T)^∗^**
CC	66	56.71 ± 7.02	102	71.65 ± 8.18	158	69.27 ± 9.10
CT	22	60.23 ± 8.30	18	69.28 ± 9.26	34	65.53 ± 11.11

*p*-value	0.049		0.209		0.030	


*In the clinical sample 120 patients had ADHD as primary or secondary diagnosis. With further 72 patients diagnosed with tic-disorders (but not with ADHD) an extended sample of 192 child psychiatry patients was also tested in a separate analysis of variance (with sex and age covariates). ^∗^At the intronic SNP only 2 children had TT genotype in the larger patient group (N = 192), who were grouped together with CT heterozygotes. Where three genotype groups were present, the minor allele carrier group was compared to the main allele homozygote group (shown in bold).*

**FIGURE 1:**
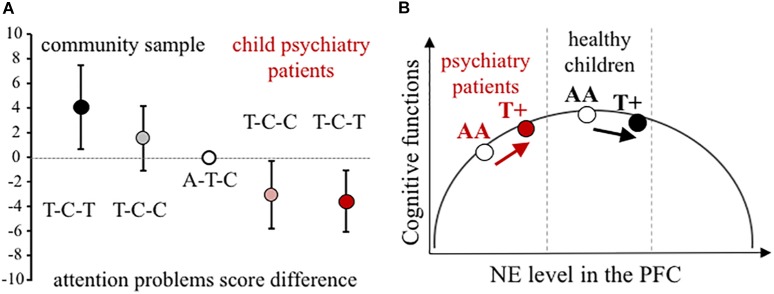
Effect of *NET* gene variants on attention processes. **(A)** Difference scores of CBCL attention problems at the *NET* haplotype groups in the community and the extended child psychiatry patient samples. Haplotypes are constructed from rs28386840, rs2242446, rs3785143 alleles. The differences in attention problems scores are presented with 95% CI of the estimated rs28386840-T ∼ rs2242446-C ∼ rs3785143-T haplotype (dark symbols, T-C-T) and T-C-C haplotype (shaded symbols) compared to the most frequent A-T-C haplotype (open circle), based on THESIAS calculations. **(B)** Proposed mechanism of the differential genetic effect of the rs28386840 (-3081) A/T SNP. The T+ group represents both AT and TT genotypes. Groups of child psychiatry patients are indicated in red.

Using all three *NET* SNPs in the estimation of haplotype effect, the rs28386840-T∼rs2242446-C∼rs3785143-T (abbreviated as T-C-T) haplotype group showed significantly higher attention problems scores in the community sample than the most frequent A-T-C haplotype group (*p* = 0.031, see 95% CI error bars above the baseline on Figure 1A). For patients with ADHD, there were no significant differences between the three most frequent haplotype groups, however, in the extended child psychiatry patient sample, both the T-C-C and the T-C-T haplotype groups showed significantly lower attention problems scores compared to the A-T-C haplotype group (T-C-C: *p* = 0.031, T-C-T: *p* = 0.005), indicating the importance of the promoter polymorphisms.

## Discussion

The involvement of the NE system in attentional networks and in ADHD pathogenesis has long been demonstrated (Ehlers and Todd, 2017; Faraone and Larsson, 2018). For example, the effectiveness of the selective norepinephrine transporter inhibitor atomoxetine was shown in ADHD treatment (Hazell et al., 2011). Furthermore, since the availability of dopamine transporter is low in the cortex, but NET is relatively abundant and can take up extracellular dopamine (Moron et al., 2002), imbalances in NET expression may contribute to attention problems due to suboptimal cortical catecholamine (both dopamine and NE) functioning.

Previously, we reported genetic associations between *NET* gene polymorphisms and inattention symptom severity on the ADHD-Rating Scale among ADHD patients (intronic rs3785143-T and promoter rs2242446-C allele carriers showed lower inattention scores, Angyal et al., 2018). Our haplotype analyses indicated that a combination of three SNPs from the 5′ end of the *NET* gene, namely the rs28386840-T∼rs2242446-C∼rs3785143-T haplotype group had significantly different score compared to the most common A-T-C haplotype group. These associations were now supported in the same group of ADHD patients using different, mother-reported questionnaire data (Table 1). In order to test genetic markers in the full range of attention (dis)functioning, we extended our analyses to healthy preschoolers recruited from the general population. The associations observed in this community sample, however, were in the opposite direction (Table 1), indicating that the underlying mechanisms may be more complex. Since other quantitative analyses of *NET* polymorphisms and attention problem scores reported mostly non-significant differences among ADHD patients (Joung et al., 2010; Park et al., 2012; Sengupta et al., 2012) and in a community sample (Hohmann et al., 2015), it remains to be seen if our genetic findings could be supported by replication studies.

Based on the inverted-U shape effect of NE (first described by Gold and van Buskirk, 1978, for more details see Arnsten, 2009), we propose that the *NET* rs28386840-T∼rs2242446-C∼rs3785143-T haplotype and/or the functional rs28386840 (-3081) T-allele have differential effects on attentional performance (Figure 1B). The -3081 T-allele showed reduced transcriptional efficiency *in vitro* (Kim et al., 2006), potentially resulting in relatively higher catecholamine levels in cortical areas. However, we have to note that an *in vivo* study using positron emission tomography to measure subcortical NET levels in adult ADHD patients and controls showed opposite effect of the -3081 T-allele in the thalamus of control subjects (no difference in NET density was observed among ADHD patients by the *NET* promoter genotypes, Sigurdardottir et al., 2016). Unfortunately, cortical areas could not be measured in this study, leaving the question open if either SNP could affect *NET* expression in the cortex.

We acknowledge that attention problems have multiple components, and genetic variants contributing to cortical *NET* expression would represent only a small part in attention processes. Since genetic factors potentially interact with each other and with environmental factors, the differential susceptibility model was tested (for more details on the model, see Belsky and Pluess, 2009). According to this model, the *NET* promoter polymorphism(s) could act as plasticity allele(s), resulting in opposite effects in positive and negative environments (reflecting that psychiatry patients have more stressful life events). In order to test this hypothesis, interaction analyses were performed in the community sample, where (mother-reported) stressful life events data was available. Since no significant interaction of life stress and genotype was observed, we rejected this model.

In conclusion, our results showed opposite genetic effects of *NET* promoter polymorphisms on attentional problems in a community sample of children compared to patients recruited at a child psychiatry clinic. The inverted-U shape modulatory effect can explain the observed contradictions if lower baseline cortical catecholamine levels are assumed in ADHD patients (see Figure 1B). According to earlier reports, disturbance of both dopamine and NE can be hypothesized in the background of ADHD (Oades, 2002). For example, measures of blood and urinary NE metabolite 3-methoxy-4-hydroxyphenylglycol indicated lower NE functioning in ADHD children (Hanna et al., 1996; Halperin et al., 1997; Llorente et al., 2006), although comorbid conditions can change the ratio of dopamine/NE (Halperin et al., 1997; Oades and Müller, 1997). Therefore, further studies are required to reveal the exact nature of neurotransmitter imbalances in ADHD in order to draw a more precise model for our *NET* genetic findings.

Limitations of our study include the relatively small sample size, which did not allow testing gene-gene interactions, and the high comorbidity rates in the clinical sample, thus it cannot be regarded as a purely ADHD patient sample. In addition, due to potential rater bias and cultural effects on the attention problems scale of the CBCL (Crijnen et al., 1999), our findings should be replicated in other cultural settings and/or with teacher- or self-report data. Future studies should also test adult patients and control subjects to see if this differential noradrenergic genetic effect is stable over time.

## Author Contributions

ZN conceived and managed the genetic association study and drafted the manuscript. NA carried out the genotyping and helped in manuscript preparation. ZT and EBo collected the questionnaire data at the child psychiatry clinic. EBi and KL collected the questionnaire data in the longitudinal study of healthy children. KL was responsible for data management and analyses of the community sample. MS-S and JG designed the data collection and acquired the financial support for it. All authors have reviewed and approved the manuscript.

## Conflict of Interest Statement

The authors declare that the research was conducted in the absence of any commercial or financial relationships that could be construed as a potential conflict of interest.
